# Damage Assessment in Rural Environments Following Natural Disasters Using Multi-Sensor Remote Sensing Data

**DOI:** 10.3390/s22249998

**Published:** 2022-12-19

**Authors:** Shiran Havivi, Stanley R. Rotman, Dan G. Blumberg, Shimrit Maman

**Affiliations:** 1 Geography and Environmental Development, Ben-Gurion University of the Negev, Beer Sheva 8410501, Israel; 2 School of Electrical and Computer Engineering, Ben-Gurion University of the Negev, Beer Sheva 8410501, Israel; 3 Homeland Security Institute, Ben-Gurion University of the Negev, Beer Sheva 8410501, Israel

**Keywords:** damage assessment, InSAR, multi-hazard, multi-sensor, rural, urban, vegetation

## Abstract

The damage caused by natural disasters in rural areas differs in nature extent, landscape, and structure, from the damage caused in urban environments. Previous and current studies have focused mainly on mapping damaged structures in urban areas after catastrophic events such as earthquakes or tsunamis. However, research focusing on the level of damage or its distribution in rural areas is lacking. This study presents a methodology for mapping, characterizing, and assessing the damage in rural environments following natural disasters, both in built-up and vegetation areas, by combining synthetic-aperture radar (SAR) and optical remote sensing data. As a case study, we applied the methodology to characterize the rural areas affected by the Sulawesi earthquake and the subsequent tsunami event in Indonesia that occurred on 28 September 2018. High-resolution COSMO-SkyMed images obtained pre- and post-event, alongside Sentinel-2 images, were used as inputs. This study’s results emphasize that remote sensing data from rural areas must be treated differently from that of urban areas following a disaster. Additionally, the analysis must include the surrounding features, not only the damaged structures. Furthermore, the results highlight the applicability of the methodology for a variety of disaster events, as well as multiple hazards, and can be adapted using a combination of different optical and SAR sensors.

## 1. Introduction

A comprehensive, rapid, and accurate damage assessment following a disaster is crucial in planning an emergency response (ER) to save lives, address the needs of the affected population, and reduce humanitarian crises [1]. However, information regarding the extent and severity of damage caused by natural disasters is often limited. Therefore, satellite data are used to assess changes after an event in a given geographical area [2]. Such images are used to compile hazard maps and rapidly provide damage assessment with high accuracy and broad spatial coverage at relatively low cost.

Amongst major disasters, earthquakes are characterized as being the most unpredictable and have a high frequency of occurrence [3,4,5]. Urban regions tend to have high population concentrations, densely situated buildings, and developed economies relative to national rural areas. They are characterized by rapid development; thus, the effect of earthquakes is potentially devastating, especially in areas where building codes have not been designed for earthquakes [1,6]. Therefore, most past and current studies have focused mainly on the mapping of damaged structures in urban areas [1,2,3,7,8,9,10,11,12]. Research focusing on the level of damage and its spatial distribution in rural areas is lacking. Moreover, in the few studies that focused on the influence of natural disasters in rural areas, the main emphasis was on damage to buildings [13,14,15].

The damage in rural areas differs in nature, both in landscape and structure, from that in the urban environment. To properly apply an emergency response, it is necessary to understand the nature of the damage in different environments.

A rural settlement generally refers to an area of open country and small settlements [16] that primarily rely on natural resources and agriculture for occupation. According to the UN, nearly half of the world’s population lives in rural areas and this proportion is expected to rise [17]. However, there is no universally accepted definition of the term ‘rural’. Rural settlements are defined by every country’s national government [18,19,20] and can vary over time. They are commonly based on population size and density (usually referred to as low population), the predominant form of economic activity (mostly agricultural), and the nature of the local infrastructure. The range and ambiguity of definitions of the term ‘rural’ are well illustrated by comparing the definitions of the term ‘urban’ (the complement of ‘rural’) [16,18].

Another definition of ‘rural’ is described by the area’s geographical and physical characteristics. Rural areas have a wide variety of land cover and land uses (LCLU), both natural and made-man, such as agricultural land, forests, and natural areas [20,21,22].

Disaster events in rural areas occur more frequently than those affecting urban areas, partly because they involve much larger areas within earthquake-prone regions [21] or because urban clusters tend to avoid disaster-prone areas or are subject to building codes relevant to local disaster probabilities. Furthermore, some disasters can trigger secondary events, such as earthquakes, triggering landslides in mountainous areas where most settlements are rural [21].

In rural areas, houses are more vulnerable to disasters and characterized by low seismic capacities because of local construction materials (such as brick, cement, wood, clay, sand, and stone) [13,23]. According to ref. [23], the collapse of houses is the main cause of casualties and property loss when a destructive earthquake occurs, causing damages to be larger. Indeed, rural buildings accounted for approximately 85% of the houses that collapsed during the Wenchuan earthquake (magnitude 8) on 12 May 2008. In addition, 90% of houses collapsed during the Yushu earthquake (magnitude 7.1) on 14 April 2010 [23].

Given that most ER research is dedicated to urban environments using specific sensors and algorithms, the novelty of this study is twofold. First, it focuses on characterizing and assessing the nature of the damage in rural regions (including built-up and vegetation areas) caused by any natural disaster. Second, this study proposes an improvement to the rapid and accurate damage assessment mapping algorithm, adapted using a combination of various optical and SAR sensors as opposed to sensor-specific algorithms.

## 2. Materials and Methods

In this section, we first present the methodology for the proposed algorithm. In two stages, we describe how the method was adapted to the characteristics of the rural areas. Initially, the existing damage assessment methodology for urban areas was modified and adjusted to rural areas. Next, damage to vegetation areas, the main LULC in the rural environment, was analyzed. As a case study, this approach is demonstrated in rural and urban environments by applying it to the Sulawesi earthquake and the subsequent tsunami and liquefaction events that occurred in Indonesia in 2018.

### 2.1. Methodology

**Figure 1:**
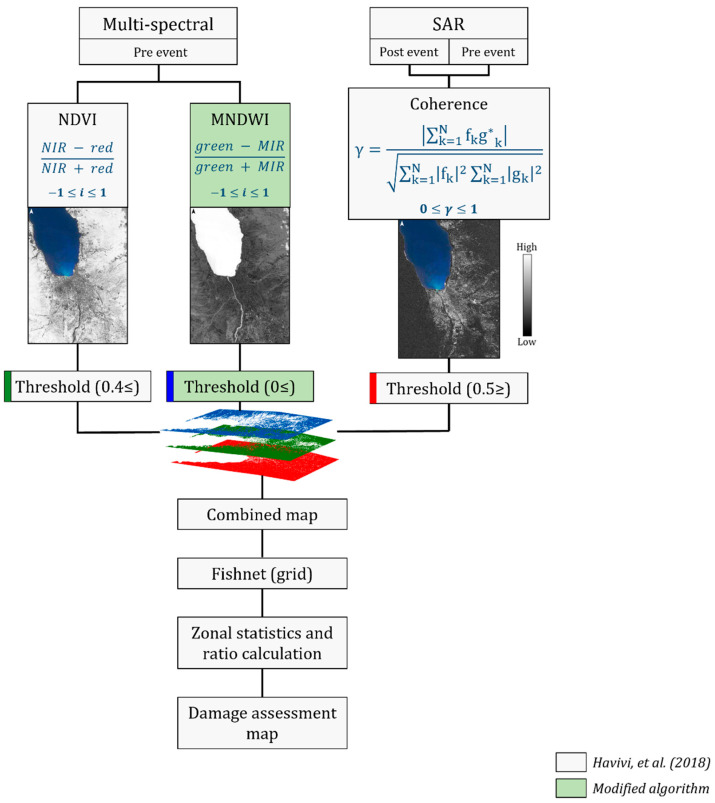
Algorithm outline. Grey blocks represent the original algorithm, proposed by ref. [1]. Green blocks represent the steps inserted to the modified process. The coherence (*γ*) is calculated by complex SAR images: *f_k_* and *g_k_*; f_k_g*_k_ is the expectation value operator. *N* represents the estimation window size in the azimuth and range directions. Light shades in the maps represent high values, and dark shades represent low values. Light shades in NDVI and MNDWI represent vegetation areas and water bodies, respectively. Light shades in the coherence map represent stable areas, and dark shades represent changed areas.

Havivi et al. [1] proposed a damage assessment algorithm aimed at rapidly mapping the damage in urban environments following earthquakes. The algorithm uses combined SAR and optical images, following the outline in Figure 1.

First, the affected areas were detected, and changes were measured by producing an interferometric synthetic-aperture radar (InSAR) coherence map [1]. In parallel, optical images were used to produce a mask by computing the normalized difference vegetation index (NDVI) and removing the vegetation area from the scene. This stage was applied to overcome the loss of coherence attributed to vegetation changes.

In rural areas, the geomorphology settings and nature differ from those of urban settlements; thus, unique changes, such as liquefaction, must be accounted for. The algorithm was modified and adjusted by inserting the modified normalized difference water index (MNDWI) [24]. This index identifies and extracts water bodies (such as streams, rivers, lakes, etc.) before the event to allow their removal, which causes a lack of coherence. Moreover, these pre-identified water bodies may further serve as indicators to highlight regions prone to being affected by natural hazards.

Once the results for coherence, NDVI, and MNDWI were obtained, thresholds were determined accordingly: ≤0.5 for the co-event coherence map [25], ≥0.4 for the NDVI [1], and ≥0 for the MNDWI [8], respectively. However, the threshold values varied according to the regional characteristics. For example, for a drier climate zone, the NDVI threshold will be lower (<0.2). Here, thresholds were determined based on the study area characterized (tropical climate) and previous studies [1,8,25]. Then, the coherence binary map was overlaid and masked by the NDVI and MNDWI threshold layers, and the three layers were combined into a new layer.

Based on the combined map of the three thresholds, coherence change detection (CCD), NDVI, and MNDWI, a damage assessment map was generated using GIS tools (Fishnet and Zonal statistics) [26]. The map grid cell size was determined according to the dominant terrain in the study area; therefore, when the topography was complex, smaller cells were used, and vice versa. Finally, the damage level for each grid cell was calculated (the ratio between the damaged area and grid cell) and expressed as a percentage per unit area.

**Figure 2:**
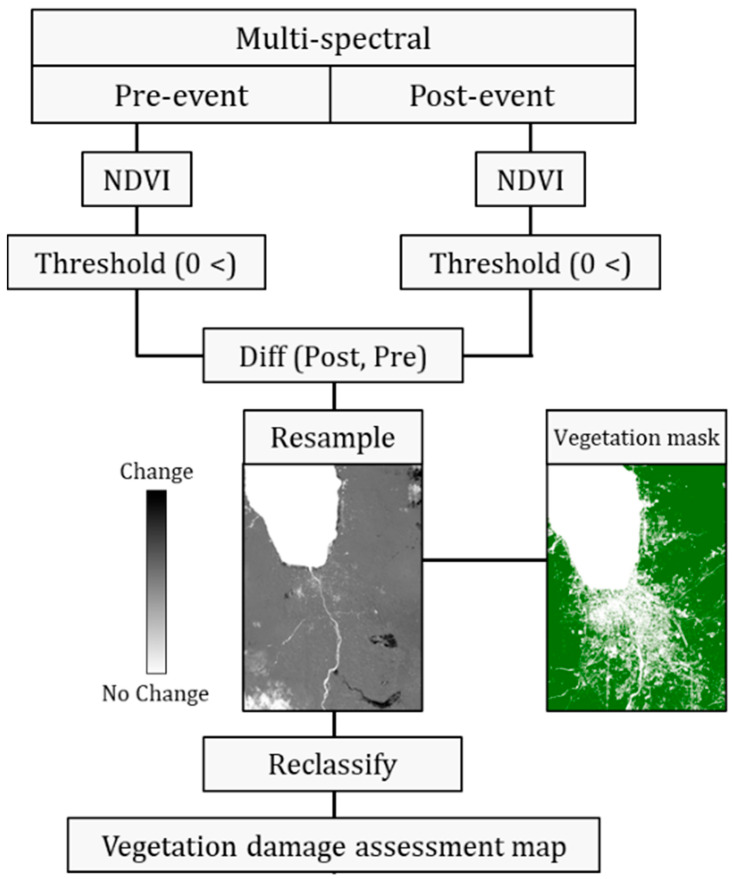
Vegetation damage assessment process outline. Light shades in the difference map represent stable vegetation areas with no or slight changes and dark shades represent vegetation areas with changes.

As mentioned above, rural areas have a wide variety of LCLU, both natural and made-man. Therefore, it is necessary to assess the damage to vegetation areas (such as cropland and grassland). For this purpose, the algorithm was modified and adjusted by inserting an additional change detection process based on the commonly used NDVI. The spatial extent of the affected vegetation areas was assessed according to the outline in Figure 2. First, the NDVI was applied to the pre- and post-event optical images. As negative NDVI values indicated non-vegetation areas, a threshold of NDVI > 0 was used to mask non-vegetation areas. The difference between post- and pre-event NDVI thresholds was then calculated. The obtained subtraction product was resampled according to the vegetation mask pixel size (previously created, Figure 1) and then extracted accordingly. The final product results were reclassified into three damage levels: (1) no damage (zero and positive values), (2) very slight damage (high negative values, 0> and ≥−1), and (3) severe damage (lower negative values, <−1).

Although a variety of sensors are available and theoretically provide pre-and post-event images, objective limitations such as temporal and spatial coverage and cloud cover prevent us from exploiting their full potential. In ref. [1], TerraSAR-X and Landsat 5 images were used. In this study, by modifying the algorithm and considering the lack of available imagery, we tested the algorithm using both different sensors and tested two suits of imagery: (1) medium spatial resolution (MSR) and (2) high spatial resolution (HSR) sensors (Table 1).

### 2.2. Data Acquisition and Processing

High spatial resolution (HSR) imagery and medium spatial resolution (MSR) imagery covering the research area were acquired before and after the earthquake and tsunami events (Table 1). The HSR images were acquired from the COSMO-SkyMed (2 m/pixel) and Sentinel-2 (10 m/pixel) satellites. MSR images were acquired from Sentinel-1 (12 m/pixel) and Landsat-8 (30 m/pixel) satellites.

The SAR images included two high-resolution X-band (3.1 cm) pre- and post-event COSMO-SkyMed images and two low-resolution C-band (5.6 cm) Sentinel-1 images. Both SAR datasets were single-look complexes (SLC) in the descending orbit direction. COSMO-SkyMed data were acquired in StripMap HIMAGE mode with a single HH polarization, an incidence angle of 28°, pixel spacing of 3 m on the vertical axis, and 3 m on the horizontal axis. Sentinel-1 data were acquired in interferometric wide (IW) swath mode with dual VH polarization, an incidence angle of 34°, a pixel spacing of 14 m on the vertical axis, and 2.3 m on the horizontal axis.

Each image was preprocessed according to the specific properties of each sensor. Precise co-registration was conducted on the COSMO-SkyMed and Sentinel-1 images, with an RMS error of up to 0.1 pixel [25]. The 23 September 2018 imagery was selected as the reference image (‘master’) for the HSR, and the 7 June 2018 imagery was selected for the MSR.

Multispectral pre- and post-event images were captured by Landsat 8 Operational Land Imager (OLI) with a spatial resolution of 30 m/pixel and Sentinel-2 (MSI level 2 B) with a spatial resolution of 10 m/pixel. NDVI was calculated based on red and near-infrared light (Figure 1): bands 4 and 8 for Sentinel-2 and bands 4 and 5 for Landsat 8. MNDWI was calculated based on green and middle infrared (MIR) light (Figure 1): bands 3 and 11 for Sentinel-2 and bands 3 and 6 for Landsat 8 (Table 2). Lastly, the Sentinel-2 and Landsat-8 images were resampled (3 m and 12 m, respectively) according to the SAR reference image (‘master’).

### 2.3. Sulawesi Earthquake Analysis

The unique K-shaped Sulawesi Island (eastern Indonesia) is located at the triple junction where the Philippine, Australian, and Sunda (the eastern part of Eurasia) plates converge [27,28,29]. It is a seismically active region where earthquakes are predominantly observed in Central Sulawesi and offshore North Sulawesi [27]. The Palu–Koro Fault (PKF) is one of the major regional active faults in Central Sulawesi, consisting of a series of strike–slip faults dominated by left-lateral movement, which generally trends NNW–SSE and N-S [27,28,30]. It lies along the west side of the Palu River, and another fault lies along the east side [31]. This long PKF trace is over 200 km (from Donggala in the north to the Palu basin) and its relative motion is estimated to be 42 mm year^−1^ [27,28,30].

The Palu Valley is situated in a narrow pull-apart basin surrounded by high mountains in the east and west, reaching an elevation of 2.3 km [27,29,31,32]. Bounded by mountains, the Palu River lowland is covered mainly by young and old alluvial fan deposits with a shallow water table [27,30,31]. Land use on the east side of the Palu Valley consists of a mixture of rice paddies and residential and commercial buildings [32]. Local rice paddies are irrigated using a system of agricultural irrigation channels [31,33,34].

This research area was chosen for several reasons. The first reason is data availability. Multiple earth observation data (SAR and multispectral data) were available for the 2018 Sulawesi earthquake and the tsunami event with high temporal resolution. Most of the data corresponded to freely accessible datasets (Sentinel-1, Sentinel-2, and Landsat 8). The second reason is that this event was characterized as “multi-hazard” (earthquake, tsunami, liquefaction, and flow slides). The final reason is that this region consists of urban and rural settlements and natural areas, including a variety of LULC, among which different damage might be observed.

**Figure 3:**
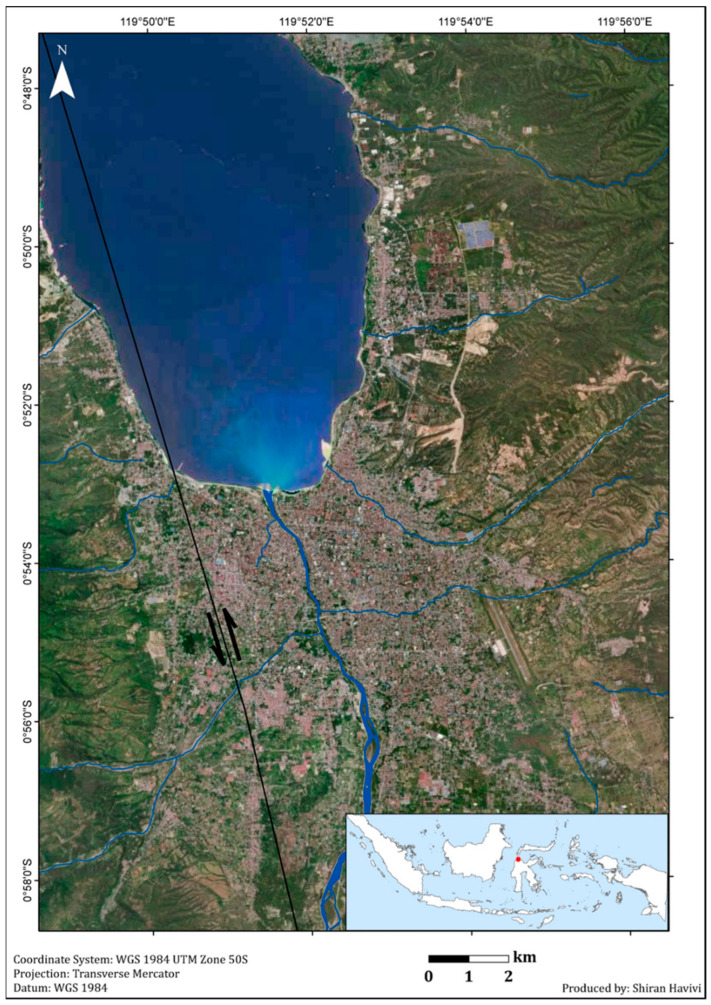
Research area: Palu, Central Sulawesi Province, Indonesia. Pre-event Sentinel-2 image (in visible bands 2—blue, 3—green, and 4—red) from 27 September 2018. The black line represents the Palu-Koro Fault (PKF). The black arrows show the dominate left-lateral movement of PKF, with trend of NNW–SSE and N-S.

The proposed algorithm described above (Chapter II) was tested on rural and urban areas affected by the Sulawesi earthquake that occurred on 28 September 2018. This earthquake, with a magnitude of 7.5, struck Central Sulawesi (Sulawesi Tengah) Island, Indonesia [35]. Its epicenter was located approximately 80 km north of Palu city (the capital of Central Sulawesi Province) (0.256° S/119.846° E; Figure 3) [36]. The earthquake triggered a destructive tsunami with a wave height that exceeded 6–11 m along the coast of Palu within Palu Bay [35], as well as landslides, soil liquefaction, and flow slides [37], and completely buried two villages (Balaroa and Petobo) [38]. This earthquake caused loss of human life and massive damage to structures and infrastructure in the area [38,39]. The extensive damage assessed and reported by the National Board for Disaster Management (BNPB) of Indonesia [40] included nearly 70,000 damaged houses.

## 3. Results and Discussion

The results presented in this section are based on the HRS datasets COSMO-SkyMed and Sentinel-2. The results based on the MRS dataset are presented in Appendix A.

**Figure 4:**
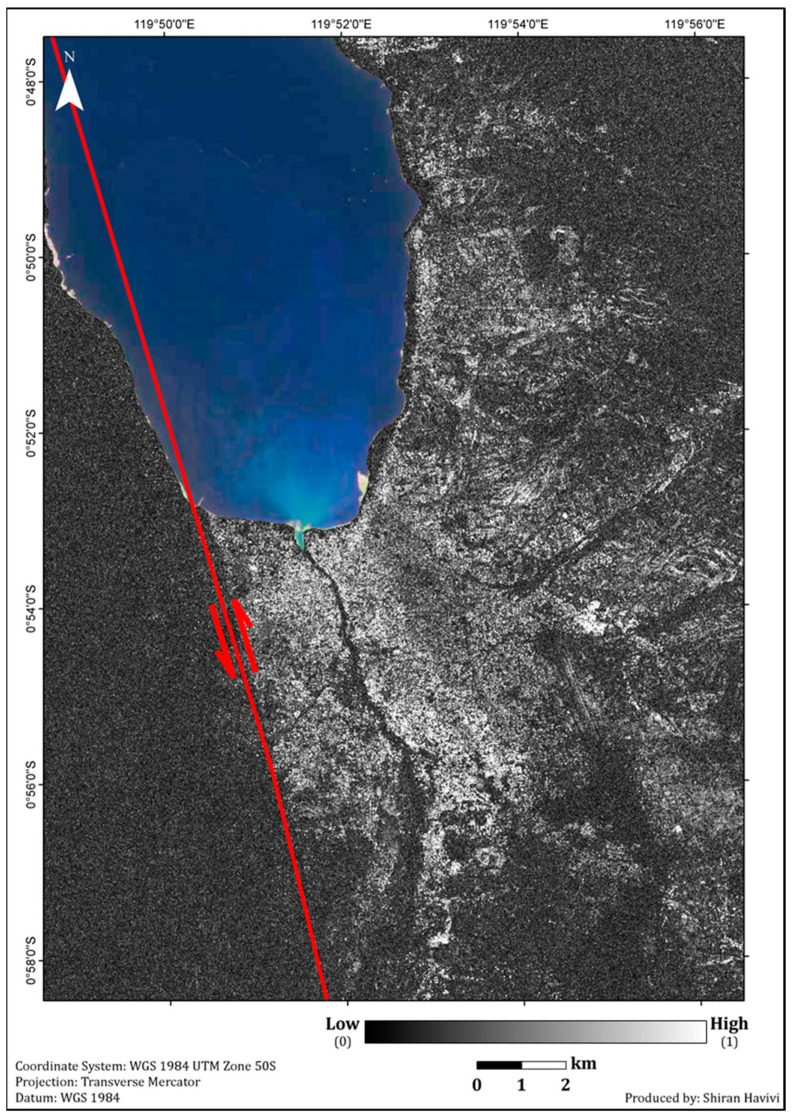
InSAR coherence map. Light tones (high coherence values) represent no change. Dark tones represent changes (low coherence values). The red line represents the Palu-Koro Fault. The red arrows show the dominate left-lateral movement of PKF, with trend of NNW–SSE and N-S.

**Figure 5:**
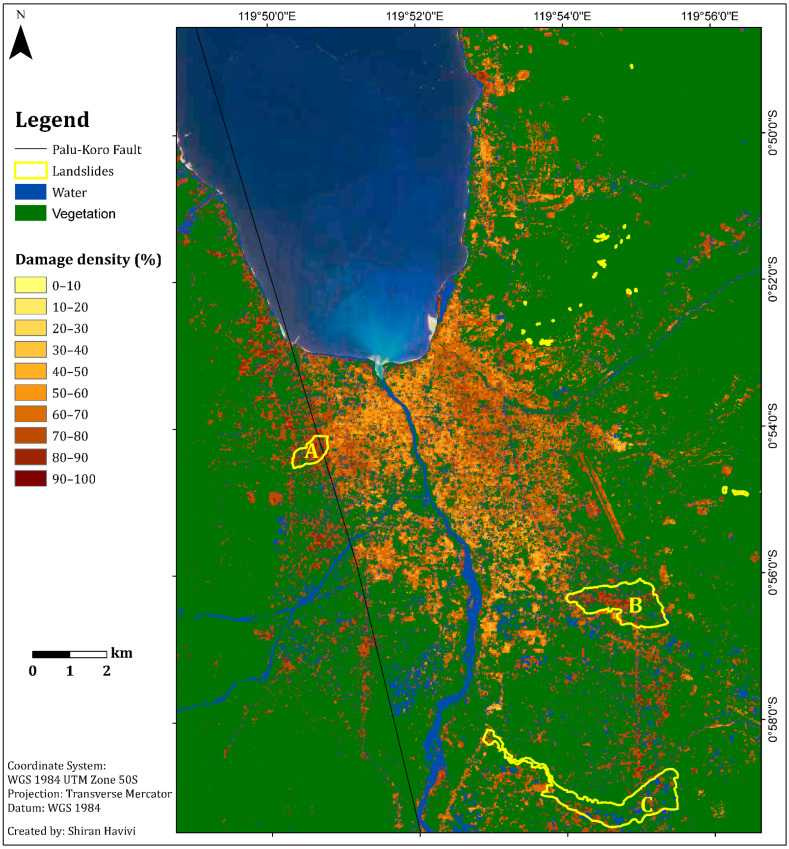
Damage density map per unit area of 50 m. Light colors represent a lower probability of damage, dark colors represent a higher probability of damage. Green represents vegetation. Blue represents water bodies. The black line represents the Palu-Koro Fault. Flow slide areas are outlined in yellow: (**A**) Balaroa (**B**) Petobo, and (**C**) Jono Oge.

The main objective was to obtain a rapid damage assessment map using the algorithm described in Figure 1. The results of the InSAR coherence and the damage density map are shown in Figure 4 and Figure 5, respectively. The NDVI and MNDWI interim results are presented in Figure 1 and were integrated according to the thresholds provided above (Figure 1).

The InSAR coherence map (Figure 4) showed a high level of change (dark shades) along the entire coastline and Palu River. Moreover, the coherence map showed a vast area that experienced changes, especially in the western part of the rupture line (PKF), which was mostly characterized by vegetation cover. The high coherence values (white tones) were attributed to the built-up area east and northeast of the fault line. The PKF is shown, stretching from the southwestern coast to the south of Palu City. Previous research has shown that the fault line results from co-seismic horizontal displacement (N-S component) of 4–7 m [27,28,29,30]. Areas east of the rupture moved to the north, and areas west of the rupture moved towards the south [27,29,30]. Due to the SAR high resolution images, reliable coherence accuracy could be achieved by calculating a small window size. This window size contained enough pixels to ensure minimal loss of information while providing a high-contrast coherence image [1].

Figure 5 shows the damage assessment map with a cell grid size of 50 m produced by the integration of three threshold maps: InSAR coherence, NDVI, and MNDWI maps. This map (Figure 5) provides information about the zones affected by earthquake and tsunami events, as well as the following liquefication. A damage density of 100% indicates total destruction. Dark colors represent a high probability of damage, whereas light colors represent a lower probability of damage [1]. Green represents vegetation areas, and blue represents water bodies.

**Figure 6:**
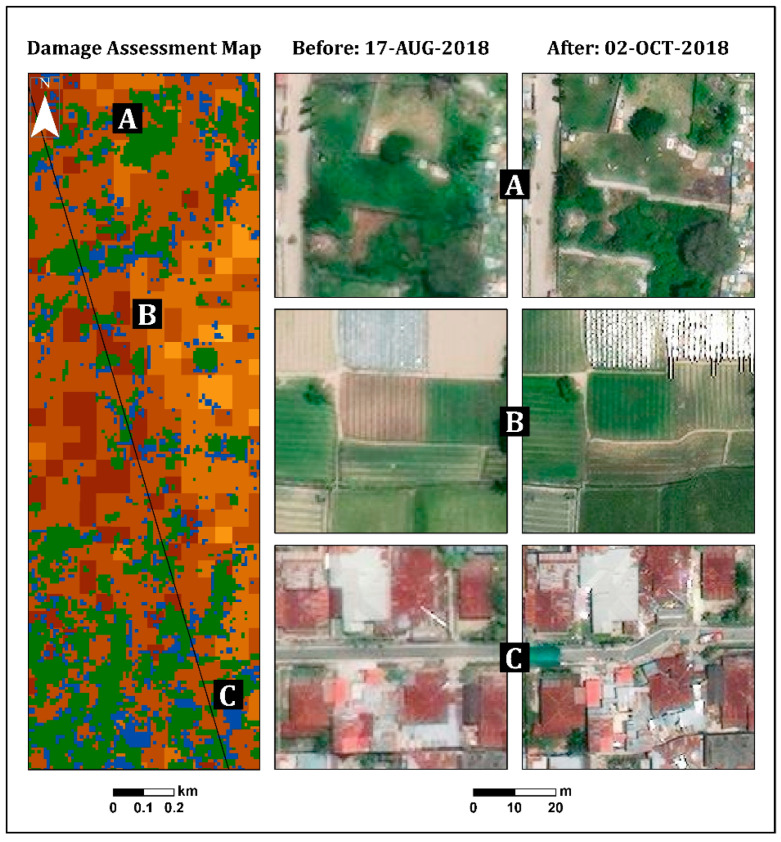
Illustration of the surface rupture caused by the 2018 Sulawesi Indonesia earthquake in (**A**) natural vegetation, (**B**) agricultural fields, and (**C**) a built-up area.

According to the damage assessment map (Figure 5), along the fault line up to a distance of 150 m (this was measured manually by visual assessment), a high level of damage (dark shades) was clearly observed from both sides, east and west (Figure 5 and Figure 6), in Palu city, and its surroundings. This high level of damage was attributed to co-seismic horizontal ground displacement (N-S component) of 4–7 m [41]. Along this fault line, three LULC scenes were examined from the east side: a built-up area, natural vegetation, and agricultural fields (Figure 6). The three insets in Figure 6, which were straight before the earthquake, show the displacement triggered by fault movement in these scenes.

**Figure 7:**
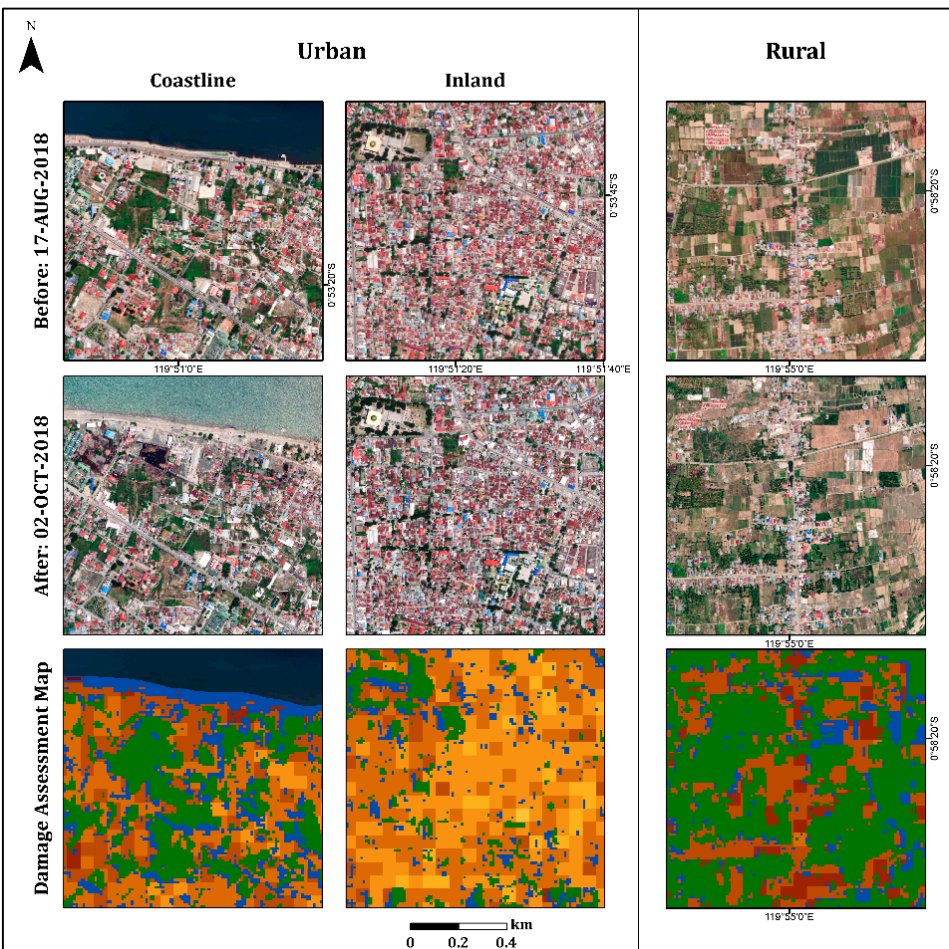
Insets showing selected urban (coastline and inland) and rural areas from the entire scene (Figure 5). Pre-event optical image (17 August 2018), post-event optical image (2 October 2018), and the damage assessment map (see legend in Figure 5).

In the urban area, the damage assessment map displayed less damage (light colors) in the inland region than in the coastal region, as expected (Figure 7). Refs. [38,39,42,43] displayed similar results, showing that most of the damage occurred along the Palu city coastline, where most of the buildings were washed away by the tsunami, and buildings inland were affected by the earthquake but not completely destroyed. Moreover, this map displayed the differences between the damage levels of the urban areas (coastline and inland regions) and the rural areas. As can be seen in Figure 7, the rural area displayed higher levels of damage (mostly darker shades) than the urban area, which displayed a variety of damage levels. These findings were further validated by high-resolution (50-cm spatial resolution) optical images recorded on 17 August (pre-event) and 2 October (post-event) 2018, using DigitalGlobe (https://www.digitalglobe.com/ecosystem/open-data/indonesia-earthquake-tsunami, accessed on 1 January 2022) (Figure 7).

**Figure 8:**
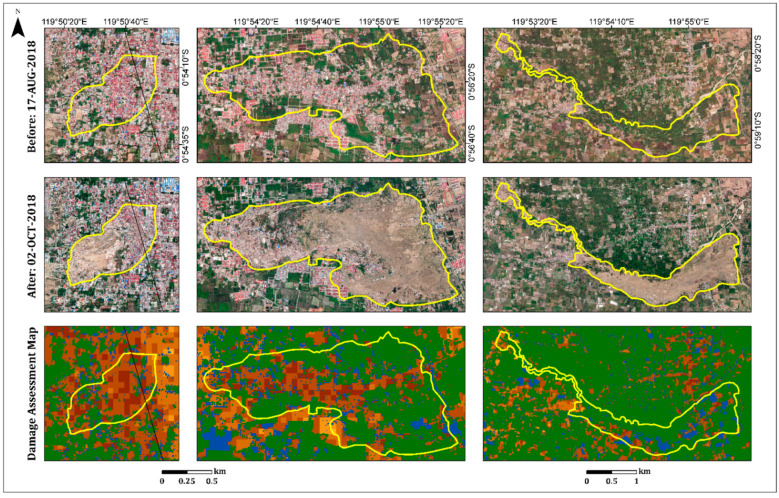
Insets showing the three villages (**left** to **right**): Balaroa, Petobo and Jono Oge. Pre-event optical image (17 August 2018), post-event optical image (2 October 2018), and the damage assessment map (see legend in Figure 5).

As a secondary hazard, the earthquake and subsequent liquefications triggered several long-distance flow slides in the Palu Basin. These destroyed suburban and rural areas on gently sloping alluvial fans: Balaroa in the west and Petobo and Jono Oge in the east [34,44,45] (Figure 5 and Figure 8). The landslide at Balaroa was much smaller than the eastern landslides, adjacent to the surface rupture, and not associated with a major irrigation system [28,34]. The flow slides caused significant loss or missing lives. Moreover, many buildings and infrastructures were severely damaged [44].

On the west side of the fault line, only the Balaroa village flow slide (Figure 8) was observed. A displacement of 3–5 m on the Balaroa slope triggered a landslide that turned into a mudflow [28]. This was influenced by the morphology of the area and colluvial fan topography, which was controlled by the natural shallow groundwater [31,41]. The landslide covered 0.4 km^2^, with a length of approximately 980 m. This occurred in a densely populated residential area (Figure 8) because Balaroa is close to the Palu city center. Figure 8 displays the damage assessment map for Balaroa Village. The area was mainly characterized by dark shades, with index values higher than 70%, indicating a high level of damage and/or complete destruction in most areas. According to the Indonesian National Agency for Disaster Management (BNPB), 2895 buildings in Balaroa were damaged or destroyed.

The affected area at Petobo, due to the flow slide induced by liquefaction, was approximately 164 Ha [44]. The maximum traveling distance of the flow slide exceeded 800 m. It was initiated from the eastern portion near the Gumbasa irrigation canal and flowed away towards the west. The Gumbasa irrigation canal, with a total length of 36 km, is the primary water source for paddy fields and other agricultural activities in the Palu Valley. The Gumbasa irrigation canal was constantly filled with water before the earthquake and the paddy fields were inundated with water (approximately 30–50 cm above the soil surface) for approximately 8 months of plantation time annually. Intensive irrigation systems to paddy fields from the Gumbasa Canal constantly supplied local groundwater tables under the paddy fields. Therefore, the groundwater levels in these areas were locally raised, which significantly contributed to the severe liquefaction phenomena and flow slide observed in Petobo during the earthquake.

The earthquake triggered ground slippage, causing cracks and drainage of the Gumbasa Canal. The northern section of the irrigation canal was completely swept away during the sliding. Moreover, land use changes were caused by earthquakes in the Petobo area. Before the earthquake, the eastern and southern parts of the Petobo flow slide area were mostly paddy fields, whereas residential areas were densely populated in the western and middle parts. According to the Copernicus Emergency Management Service (Copernicus EMS), a flow slide damaged or destroyed more than 3000 buildings in Petobo. Therefore, the loss of life and injuries were large. Furthermore, flow slide liquefaction contributed to changes in soil elevation in the Petobo area. The elevation dropped in the eastern part (5–8 m) and increased (4–10 m) in the western part. In the middle part, the elevation dropped (more than 7 m) in one area and was raised (1–4 m) in another. The surface grades changed accordingly.

Figure 8 shows the damage assessment map for the Petobo area. The Gumbasa Canal is prominent on the eastern side of the flow slide boundary. The received damage (in dark shades) was clearly observed in the northern part of the flow slide. The southern part was mostly covered by vegetation; therefore, the algorithm did not show any damage in this area. However, where vegetation cover was missing, a high level of damage was observed. Pre-event imagery was used to generate the vegetation mask; therefore, the received damage to the agricultural fields, which were damaged or even destroyed by the flow slide, could not be observed. A good example is the eastern part of Petobo, next to the irrigation canal (Figure 8), which was mainly covered by agricultural fields.

The Jono Oge flow slide extended over an area of 1.35 km^2^, with a maximum displacement of 2 km [32]. It started near the irrigation canal, where the area was primarily agricultural (over 90% of the area), comprising mostly rice paddies and tomato fields, with modest population density [45,46]. However, its large footprint affected many people downslope. The flow slide destroyed one of the primary roads of Central Sulawesi, connecting the north (Palu) and south of the island, which had a significant impact on mobility and consequently, economic recovery in the region. Moreover, more than 300 residential buildings were damaged or destroyed [47]. Only one building with a heavily reinforced foundation resisted the flow slide and stayed in the same position, whereas the surrounding houses were all swept away for several hundred meters [31,46].

At the time of the earthquake, the river in the northern edge of the affected area was empty of water, yet houses along the river were inundated [31]. The flow slide breached the walls of the irrigation canal and destroyed the water gate, resulting in the discharge of a large volume of water. Failure and water from the canal drained into the slide area. The water transitioned into a debris flood that scoured within the flow slide area and traveled to downslope villages, reaching the Palu River approximately 6 km away [45]. The debris flood completely eroded or significantly altered many of the morphological features along its path [31].

**Figure 9:**
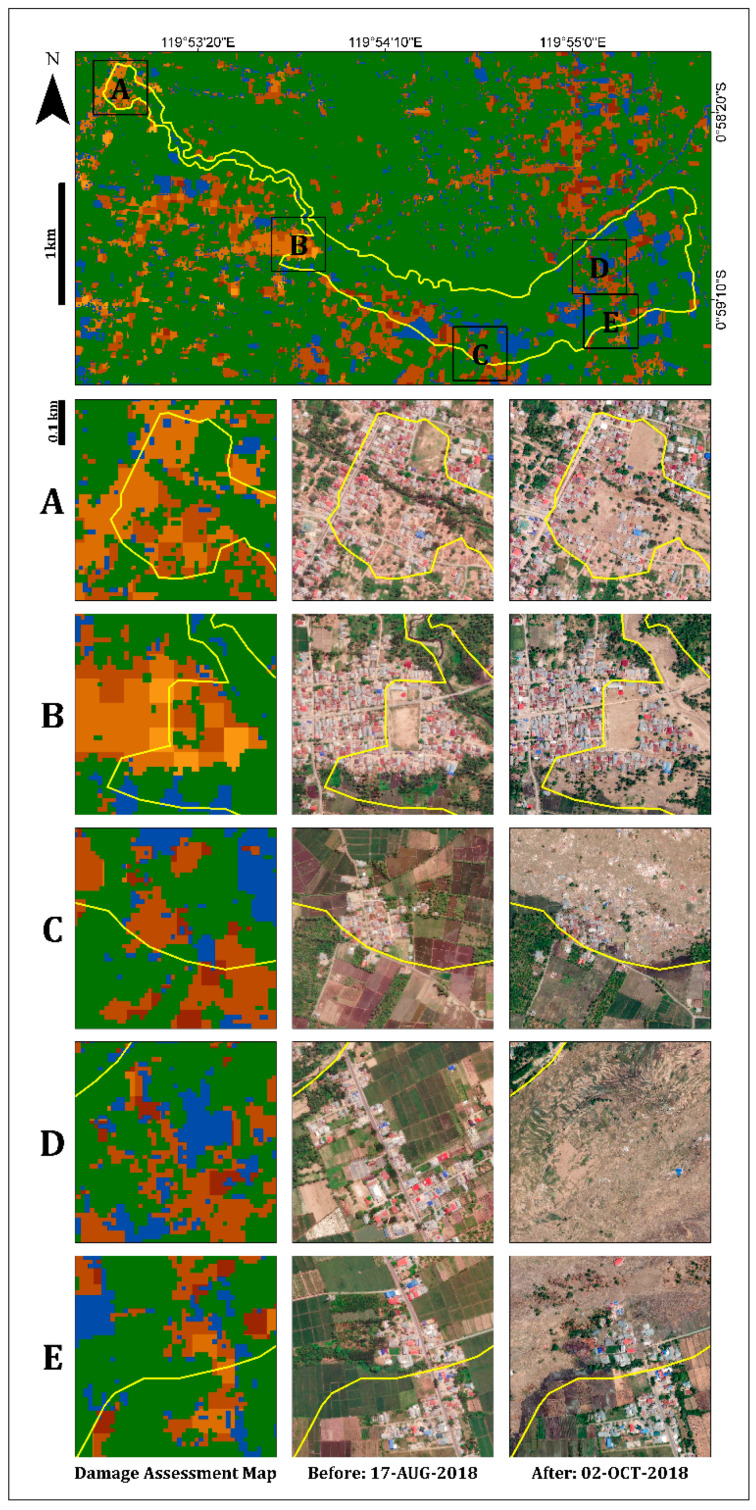
Zoomed in damaged built-up areas in Jono Oge village (subfigures **A**–**E**) resulting from the second hazards, liquefication and flow slide. Pre-event optical image (17 August 2018), post-event optical image (2 October 2018), and the damage assessment map (see legend in Figure 5).

Figure 9 displays the damage assessment map of Jono Oge Village. As previously mentioned, the primary flow slide area was agricultural. Therefore, most of the flow slide area was covered with a vegetation mask. However, a built-up area damaged by the flow slide and mudslide (the left edge) was observed. Moreover, the irrigation canal and river at the northern edge were observed, as well as the rice paddies, which could be identified as flooded. Identification of irrigation canals, stream routes, and agricultural fields assists in understanding, in a time of disaster, which areas are prone to landslides and therefore have the potential for the greatest damage.

**Figure 10:**
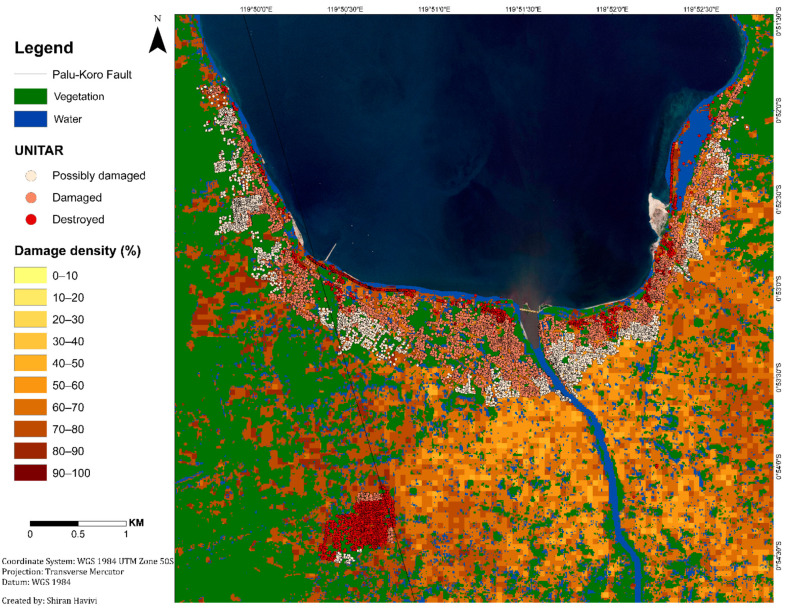
Damage assessment map vs. UNITAR database.

The algorithm results were validated using the UNITAR (https://www.unitar.org/maps/map/2855, accessed on 1 January 2022) database (Figure 10). This point shapefile database provides a building damage assessment for the entire affected area. Three damage grades were defined: possible damage (light color), damaged buildings (pink color), and destroyed buildings (red color). This damage analysis was evaluated through visual interpretation based on optical satellite imagery, with a 50 cm resolution, obtained from WorldView-2 (acquired on 1 October and 4 October 2018), Pleiades 1A/1B (acquired on 30 September and 2 October 2018), and WorldView-3 (acquired on 2 October 2018).

**Figure 11:**
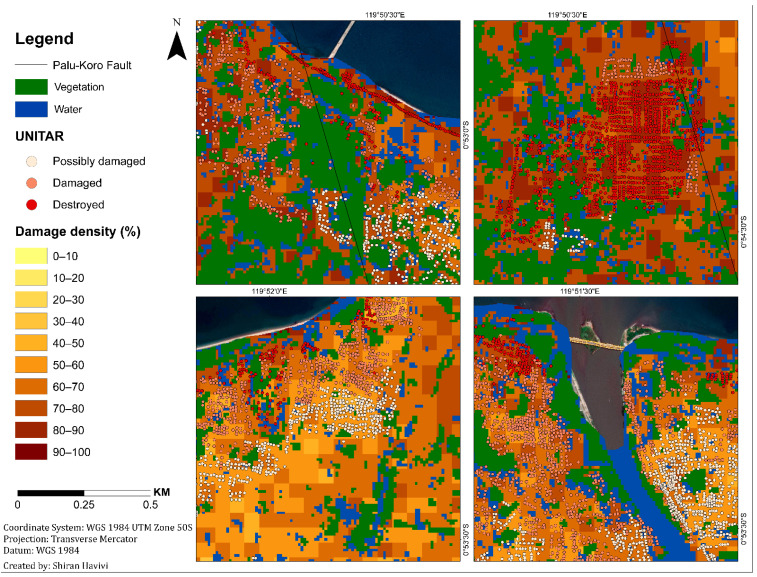
Damage assessment map vs. UNITAR database. Insets of urban and rural areas.

The correlation between the UNITAR database and damage algorithm results was calculated. It was applied using the geographically weighted regression (GWR) tool in GIS. GWR is a local form of linear regression used to model spatially varying relationships [47]. Although the two databases differed in type (point information vs. area unit), a high correlation between the two was obtained. In urban areas, the algorithm results and the UNITAR database were highly correlated (R^2^ = 80%). In rural areas, the correlation was even higher (R^2^ = 90%) (Figure 11). R^2^ values differed in rural and urban environments owing to the different densities of the structures. Rural density is lower than urban density. However, these high correlation values indicated that the presented algorithm can be easily implemented to rapidly and reliably identify and assess damage in built-up areas in rural and urban environments.

**Figure 12:**
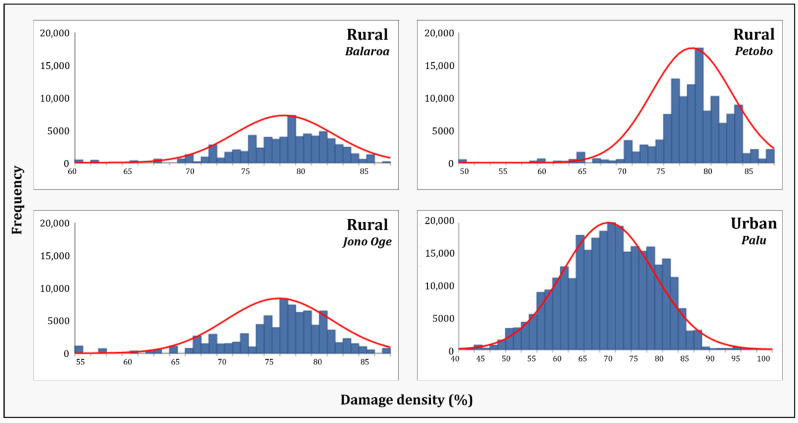
Damage level value distribution of the rural (Balaroa, Petobo, Jono Oge) and the urban (Palu) areas.

Table 3 summarizes the damage assessment results of built-up areas in urban and rural environments. None of these areas received a damage level value of zero. Thus, built-up areas in both environments experienced changes following the earthquake event. The lowest received damage level value (meaning less damage—Min) was in the urban environment (38.8). In comparison, the damage levels in rural areas started with higher values. Balaroa had the highest initial value (59.4). The highest damage level value (Max = 100) was only found in the urban environment (total destruction). The mean and median values in the urban environment were the lowest(both approximately 66). These indices were higher for the three rural areas (75–78). Likewise, the majority value in the urban environment was the lowest(65.4) and the minority was the highest (88.6). As for the rural areas, the majority values were 75–78 and the minority values were 65–80. Namely, there were more low damage values and fewer high damage values in the urban area than in rural areas. All of the histograms of the urban and rural environments (Figure 12) had negative skewness values, showing a slight tendency to the left (left-skewed distribution). Nevertheless, the distribution of the urban environment values was less spread out (Kurtosis = 2.6) compared to that of the rural environment values (5–8). These indices indicated that the urban area damage level values were almost normally distributed, showing symmetry on both sides (skewness = −0.2). These measurements indicated that built-up areas in the rural areas experienced more damage than the urban area.

While the damage in the built-up areas was observed by the algorithm in both rural and urban environments, the possibility of monitoring the damage in vegetation areas (natural and agricultural) did not exist. This is because a vegetation mask was used to eliminate the vegetation areas. Rural regions include and are surrounded by extensive vegetation areas. Therefore, changes in these areas owing to disaster events should be monitored and assessed.

**Figure 13:**
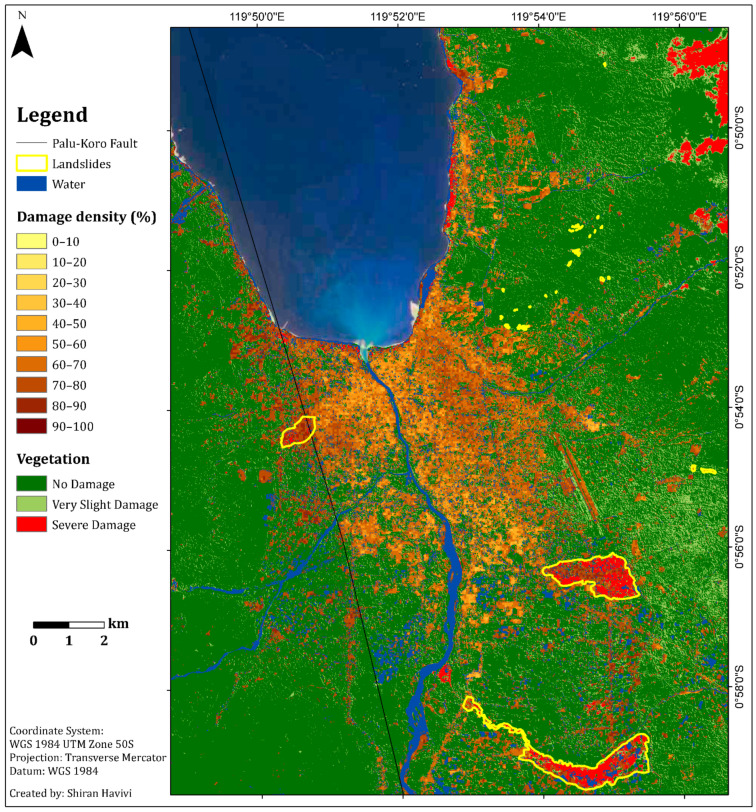
Final damage assessment map (50 m per pixel) generated using both damage assessment methods for the built-up and vegetation areas. Light colors represent a lower probability of damage, dark colors represent a higher probability of damage. Blue represents water bodies. The black line represents the Palu-Koro Fault. Flow slide areas are outlined in yellow. Dark green represents undamaged vegetation. Light green represents a slight change in vegetation. Red represents severe damage to vegetation.

Figure 13 shows the final damage assessment map. Based on the two damage assessment methods, this map was generated for built-up and vegetation areas (Figure 1 and Figure 2). The map provides a vegetation (natural and agricultural) damage assessment of the entire affected area, as well as the damage levels for the built-up areas.

The total vegetation area in this study was 287 km^2^. Following the disaster, 54 km^2^ (19%) of the vegetation area experienced a certain level of damage. This area was divided into 36.3 km^2^ of very slight damage (13%) (light green color) and 17.7 km^2^ of severe damage (6%) (red color). The vegetation areas that experienced the most significant damage following the earthquake event were located along the coast and in the village regions. These areas were damaged due to secondary geo-hazards, such as the tsunami wave along the coast of Palu and liquefaction and flow slides in the villages (Balaroa, Petobo, and Jono Oge) (Figure 13).

**Figure 14:**
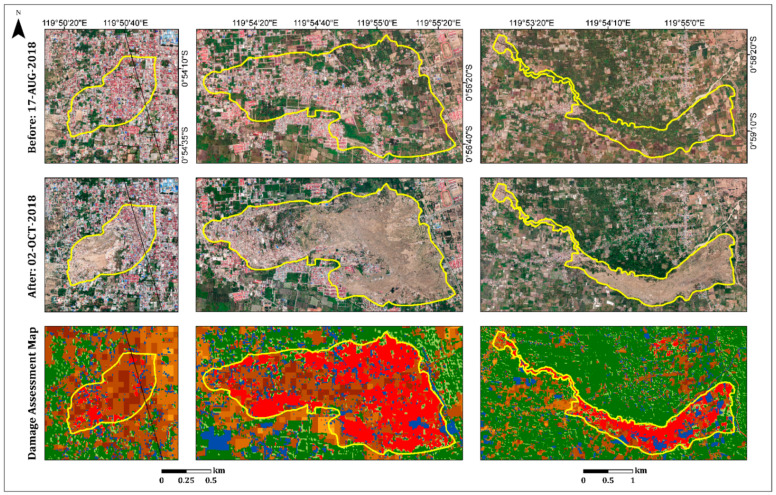
Insets showing the three villages (**left** to **right**): Balaroa, Petobo and Jono Oge. Pre-event optical image (17 August 2018), post-event optical image (2 October 2018), and the overall damage assessment map: structures and vegetation (see legend in Figure 13).

Figure 14 shows the overall damage to the three villages, Balaroa, Petobo, and Jono Oge, in the built-up and vegetation areas. Most of the vegetation in these villages was severely affected (red color) by the flow slides. No damage or very slight damage was found mostly outside the flow slide area (yellow polygons) and very little damage was found inside these areas.

Table 4 summarizes the vegetation damage in the rural areas in detail. Furthermore, the relative sizes of the built-up, water body, and vegetation areas included in these three villages are presented here. As was previously mentioned, rural areas are characterized by large vegetation areas. Balaroa has the smallest vegetation area (21%) compared to Petobo (58%) and Jono Oge (68%). This smallest vegetation area is mainly due to the village’s proximity to Palu city (Figure 13). Moreover, it can be seen that the vegetation area size increases relative to the distance from Palu city and the water body areas. Respectively, the built-up area size decreases with increasing distance.

All three villages experienced damage in their vegetation areas, mostly at a severe level. Table 4 summarizes the relative damaged vegetation area size by the damage grade. The largest severely damaged area was observed in Petobo (81%), followed by that in Balaroa (70%) and in Jono Oge (66%). The minor areas that experienced no or slight damage were in Petobo, with 9% and 10%, respectively. The other two villages, Balaroa and Jono Oge, received very slight (18% both) and no damage (11% and 16%, respectively) in larger areas.

Considering the vegetation results, it can be concluded that the NDVI threshold level should be adjusted based on the regional characteristics. The vegetation LULC variety (cropland, farmland, natural, etc.) in the rural environment is greater than that in the urban environment; therefore, successful coverage will be obtained with a lower NDVI threshold. In this study, the NDVI threshold for rural areas was changed from 0.4, which is considered appropriate for urban areas, to 0.15. This change was due to the cropland regions, which were found to be dry with low NDVI values.

In contrast with other methods requiring many high-quality training samples [48,49,50,51,52,53], the results of this study highlight the efficiency of the presented methodology by detecting changes without using training areas. Therefore, the time required for the damage assessment mapping process is reduced and the potential for implementation in other disaster events is increased. Furthermore, unlike most studies that have classified the damage into small categories, usually between two and four categories [53,54,55,56,57] focusing only on one type of object (usually in built-up areas), this study provides various damage levels for both built-up and vegetation areas.

## 4. Conclusions

The damage in rural areas differs in nature, both in landscape and structure, from that in the urban environment. To apply an ER, it is necessary to understand the nature of the damage in different environments. This study presented a new method for mapping, assessing, and characterizing the nature of the damage in rural regions following a natural disaster by combining SAR and optical remote sensing data. As a case study, this algorithm was applied to areas affected by a multi-hazard event following the Sulawesi earthquake and the subsequent tsunami in Palu, Indonesia, which occurred on 28 September 2018.

The method proposed here is an improvement and adjustment of the method described by Havivi et al. [1] for rapid mapping and compiling a damage assessment map following a natural disaster in a rural area (for built-up and vegetation regions). The output damage assessment map provides a quantitative assessment and spatial distribution of damage in both rural and urban environments. The results highlight the applicability of the algorithm for a variety of disaster events, as well as multiple hazards, and could be adapted using a combination of different optical and SAR sensors, as opposed to sensor-specific algorithms. Furthermore, this methodology can be adapted for urban and rural environments with but a few adjustments.

An analysis of the damage in rural and small settlements will contribute to our understanding of the damage following a natural disaster. Furthermore, it provides a more complete picture of the extent of the damage and access to most of the affected population.

## Figures and Tables

**Table 1:** Satellite images used in this study.

	Satellite	Sensor Type	Acquisition Date	
HSR	COSMO-SkyMed	SAR	Pre-event Post-event	23 September 2018 * 5 October 2018
	Sentinel-2	Multispectral	Pre-event Post-event	27 September 2018 2 October 2018
MSR	Sentinel-1	SAR	Pre-event Post-event	7 June 2018 * 5 October 2018
	Landsat-8	Multispectral	Pre-event	16 September 2018–23 September 2018

* Imagery was defined as ‘master’.

**Table 2:** Spectral information used for indices calculation.

Index		Sentinel-2		Landsat-8	
		Band	Range (μm)	Band	Range (μm)
**NDVI**	Red	4	0.64–0.68	4	0.64–0.67
	NIR	8	0.76–0.90	5	0.85–0.88
**MNDWI**	Green	3	0.53–0.58	3	0.53–0.59
	MIR	11	1.53–1.68	6	1.57–1.65

**Table 3:** Comparing the damage assessment results of built-up areas in rural areas vs. the urban area by summarizing the statistics.

	Min	Max	Mean	Median	Std. Dev.	Skewness	Kurtosis	Majority	Minority
*Balaroa*	59.4	86.8	77.5	78	4.3	−1.1	5.1	78	79.9
*Petobo*	48.2	87.3	77.1	77.7	5	−1.5	8	78.2	65.6
*Jono Oge*	53.9	86.6	75	75.9	5.5	−1.2	5.1	75.7	65.5
*Urban*	38.8	100	66.1	66.5	8.3	−0.2	2.6	65.4	88.6

**Table 4:** Summary of the damaged rural areas in Palu valley. Built-up, vegetation, and water body areas are included in the data. A detailed description of the damaged vegetation is provided along with the damage level.

	Area km^2^		Area%			Vegetation Damage%		
	Total	Vegetation	Vegetation	Water	Built	No Damage	Very Slight	Severe
*Balaroa*	0.43	0.09	21%	7%	72%	11%	18%	70%
*Petobo*	1.76	1.02	58%	9%	33%	9%	10%	81%
*Jono Oge*	2.24	1.53	68%	15%	17%	16%	18%	66%

**Figure A1:**
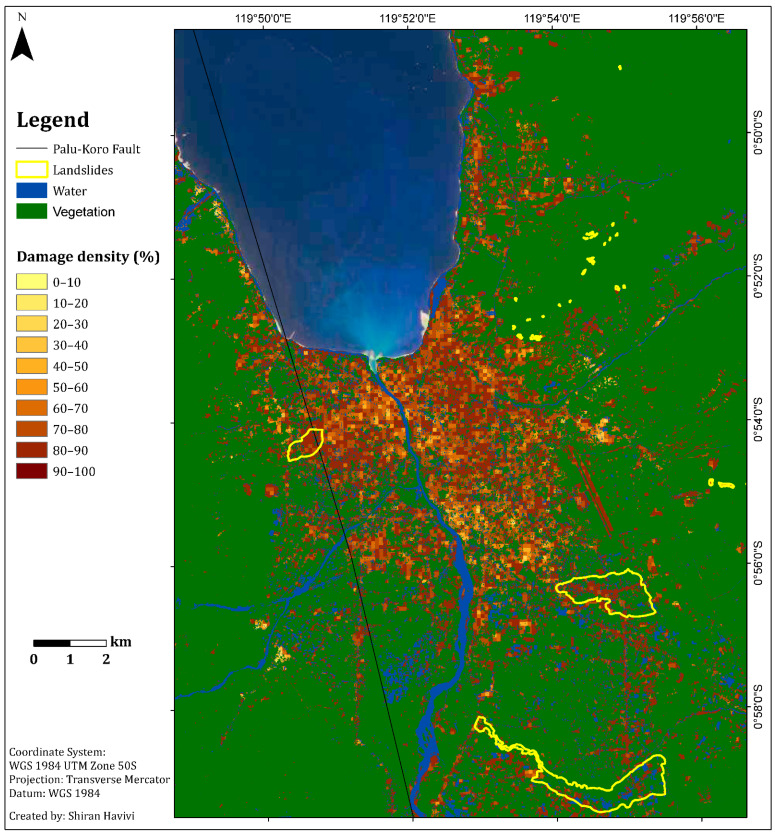
Damage assessment map generated from medium spatial resolution (MSR) images using Sentinel-1 and Landsat-8 satellites.

## Data Availability

The Sentinel-1 data (SAR) used in this study can be accessed in a publicly archived dataset https://search.asf.alaska.edu/#/, accessed on 28 September 2022, at the Alaska Satellite Facility (ASF). The Sentinel-2 and Landsat-8 data (multi-spectral) used in this study can be accessed in a publicly archived dataset https://apps.sentinel-hub.com/eo-browser, accessed on 28 September 2022, at the EO browser of Sentinel-hub.
